# Commentary on Brennan *et al*.: Pros and cons of minimum unit price for alcohol

**DOI:** 10.1111/add.16165

**Published:** 2023-03-01

**Authors:** Heng Jiang, Robin Room

**Affiliations:** ^1^ Centre for Alcohol Policy Research, School of Psychology and Public Health La Trobe University Melbourne Australia; ^2^ Department of Public Health, School of Psychology and Public Health La Trobe University Melbourne Australia; ^3^ Melbourne School of Population and Global Health University of Melbourne Melbourne Australia; ^4^ Centre for Social Research on Alcohol and Drugs, Department of Public Health Sciences Stockholm University Stockholm Sweden

**Keywords:** Alcohol tax, effectiveness, harm prevention, inequalities, local communities, minimum unit price

*A combination of a tax increase and minimum unit price for alcohol may achieve better effects on harm prevention for various drinking, age, and socioeconomic groups*.

Minimum unit price (MUP) policies for alcohol have been used to control and prevent alcohol‐related harms in several countries and regions [1, 2, 3, 4]. Although some government retail alcohol monopolies have long had minimum price levels as internal rules, in recent years, new MUP policies have been implemented in privatised markets by levels of government, which are strongly motivated to reduce levels of alcohol‐related harm in their jurisdiction, but do not have the constitutional authority to impose or raise alcohol taxes.

Whether a general alcohol tax increase or imposing a MUP will have more effect in reducing alcohol consumption and problems is likely to depend on the amount of change in retail prices, particularly of cheaper forms and brands of alcohol, which the measure imposes. The effects may vary also because of variations in drinking preferences, socio‐economic status and responses of demand to price change in different regions or localities. This means that local policies of MUP need to be informed by local evidence on the potential positive overall effect in reducing alcohol consumption and harms. The study by Brennan *et al*. [5] is a good example of providing such evidence. Brennan *et al*. [5] have modelled the effect and effectiveness of the MUP in 50 local authorities or regions in England. The modelling study found that the MUP could help to reduce alcohol's death, hospitalisations, health service costs and crime rates in various local communities in England, with greater prevention effects in the three northern regions of England compared with the national average, highlighting that the MUP policy may have great potential to reduce health inequalities in England.

This study mainly focused on the mortality and morbidity of drinkers and on crimes committed under the influence of alcohol; however, harms of alcohol use often go beyond the drinkers to the people around drinkers, such as the drinker's family, friends, co‐workers and by‐standers. For example, our study [6] found that one in three people in Australia need to spend on average 32 hours a year to look after their heavy drinking family members, friends, or co‐workers and the drinkers' dependents, costing AUD $2.9 billion (in terms of productivity loss) or 15% of the total costs to the society annually. Therefore, the measured effects of MUP in local communities could be greater if the preventive effects on alcohol's harm to others are included.

Previous studies [7, 8] have pointed out that the majority of alcohol‐related harms in the total population may occur among low‐to‐moderate risk drinkers, because they are a large proportion in the total population, although high‐risk or heavy drinkers have a higher individual risk of experiencing alcohol‐related harms. The MUP policy is likely to target those heavy or high‐risk drinkers who like to purchase cheap alcohol [9]. It may generate minor impact on light and moderate drinkers, which may mean the effects of MUP on the majority of alcohol‐related harms that occur among low‐to‐moderate drinkers is small. Furthermore, among different income groups, the effects of MUP could be varied, since high‐income heavy drinkers are less likely to be affected by the MUP, and our research in Australia also found rich middle‐aged and older drinkers have become a substantial group with alcohol related problems [10]. Given the ageing population in Australia, the United Kingdom and many other countries, this will become a major problem for controlling alcohol‐related harm, and MUP may only achieve minor impact on this specific group. In contrast, an alcohol tax increase generally applies to all population groups, and it could achieve considerable harm prevention effects on low and moderate drinkers and older age groups [11]. Therefore, a combination of tax raise and MUP should be considered, and it may achieve better preventive effects on various drinking, age and socio‐economic groups.

The MUP is a good way to control alcohol affordability, although it needs to be adjusted with the inflation annually to remain its effectiveness. However, financial gains because of the price increase (because of the MUP) will go to alcohol retailers and producers [12]; estimates for Ireland and the United Kingdom show these to be substantial [13]. Alcohol retailers may use this revenue gain to advertise alcohol more and promote alcohol use, which may partially undercut MUP's positive effect in limiting alcohol consumption. However, the positive overall effect in reducing alcohol consumption and harms found in studies of MUP's effects provide reassurance that the effects of any such increased promotion are marginal and outweighed by the positive effects.

## AUTHOR CONTRIBUTIONS

HJ and RR both drafted and wrote this paper, and contributed equally.

## DECLARATION OF INTERESTS

None.

## Data Availability

Data sharing not applicable to this article as no datasets were generated or analysed during the current study.
